# Effects of *Aronia melanocarpa* Constituents on Biofilm Formation of *Escherichia coli* and *Bacillus cereus*

**DOI:** 10.3390/molecules181214989

**Published:** 2013-12-05

**Authors:** Marie Bräunlich, Ole A. Økstad, Rune Slimestad, Helle Wangensteen, Karl E. Malterud, Hilde Barsett

**Affiliations:** 1 Department of Pharmaceutical Chemistry, School of Pharmacy, University of Oslo, P.O. Box 1068, Blindern, Oslo N-0316, Norway; E-Mails: helle.wangensteen@farmasi.uio.no (H.W.); k.e.malterud@farmasi.uio.no (K.E.M.); hilde.barsett@farmasi.uio.no (H.B.); 2 Laboratory for Microbial Dynamics and Department of Pharmaceutical Biosciences, School of Pharmacy, University of Oslo, P.O. Box 1068, Blindern, Oslo N-0316, Norway; E-Mail: aloechen@farmasi.uio.no; 3 PlantChem, Særheim Research Center, N-4353 Klepp Station, Norway; E-Mail: rune@plantchem.com

**Keywords:** *Aronia melanocarpa*, biofilm formation, *Escherichia coli*, *Bacillus cereus*, flavonoids

## Abstract

Many bacteria growing on surfaces form biofilms. Adaptive and genetic changes of the microorganisms in this structure make them resistant to antimicrobial agents. Biofilm-forming organisms on medical devices can pose serious threats to human health. Thus, there is a need for novel prevention and treatment strategies. This study aimed to evaluate the ability of *Aronia melanocarpa* extracts, subfractions and compounds to prevent biofilm formation and to inhibit bacterial growth of *Escherichia coli* and *Bacillus cereus* *in vitro*. It was found that several aronia substances possessed anti-biofilm activity, however, they were not toxic to the species screened. This non-toxic inhibition may confer a lower potential for resistance development compared to conventional antimicrobials.

## 1. Introduction

Bacterial biofilms are sessile communities embedded within a self-produced matrix of extracellular polymeric substance (EPS) that are ubiquitous in natural, medical, and engineering environments [1,2]. Biofilms formed by pathogenic Gram-negative *Escherichia coli* strains can pose serious problems to human health, such as prostatitis, biliary tract infections, and urinary catheter cystitis [3]. Deleterious biofilms are also problematic in industry since they can cause fouling and corrosion in systems such as heat exchangers, oil pipelines, and water systems [1]. *Bacillus cereus* is a Gram-positive, spore forming bacterium closely related to the human and animal pathogen *Bacillus anthracis*, the cause of anthrax. *B. cereus* is frequently identified as the causative agent of food-borne diseases. As such, the interest in this bacterium is growing. This ubiquitous organism can easily contaminate food production or processing systems and forms biofilms that are highly resistant to cleaning procedures [4,5]. In addition to food poisoning, *B. cereus* strains have the potential to cause a number of systemic and local infections in both immunologically compromised and, although less frequent, immunocompetent individuals. Certain groups of individuals are more commonly infected, including neonates, intravenous drug abusers, and patients with traumatic or surgical wounds, or with indwelling catheters. The disease spectrum is wide, including (but not limited to) catheter-related bloodstream infections, central nervous system (CNS) disease (meningitis and brain abscesses), endophthalmitis, and pneumonia [6].

Bacteria in a biofilm are often responsible for reoccuring symptoms and medical treatment failure [7,8]. Biofilm infections are difficult to eradicate because the genetic program (global gene expression pattern) of bacteria within such a structure is fundamentally changed, resulting in increased protection against e.g., macrophages and antibiotics, compared to planktonic (free living) cells [9]. Thus, the eradication of *E. coli* biofilms required 220 times higher antibiotic concentrations than for the same strain in serum. Also, the fact that many cells in a biofilm live for extended periods without going through cell division, contributes to resistance toward antibiotics, which are primarily effective on dividing cells [8]. In addition, some protection may be conferred by the physical barrier provided by the presence of the EPS that covers the biofilm and may prevent sufficient antibiotic exposure to kill the cells. Hence, novel antagonists with the potential to remove and/or prevent the formation of biofilms are needed. Agents which do not directly inhibit bacterial growth may confer a lower selection pressure for resistance development [1,10]. Recently, there has been a tremendous increase in biofilm research. An important focus of this research has been the development of alternative approaches, either to avoid the use of antimicrobials altogether, or to combine alternative treatments with more traditional antimicrobial drugs with the potential to totally eliminate biofilm formation on e.g., indwelling devices [11].

The aronia plant (*Aronia melanocarpa* (Michx.) Elliott var. Moscow (Rosaceae)) has gained popularity in recent years due to its berries with a high content of polyphenols with antioxidant activity [12,13]. Aronia products are used as nutritional supplements, and the berries are an important source for juices, wines, and jams, and constitute a rich source of natural food colorants [12,14]. Aronia berries contain high levels of flavonoids, mostly proanthocyanidins and anthocyanins. Also, chlorogenic and neochlorogenic acids are known constituents [15].

Here we present the first study investigating the effects of aronia constituents on biofilm formation, complementing earlier studies investigating the antimicrobial activity of aronia berry extracts against *Staphylococcus aureus*, *Escherichia coli*, and type A influenza virus [16]. The aim of the present study was to screen extracts, subfractions and compounds from *A. melanocarpa* for their abilities to inhibit biofilm formation of *E. coli* and *B. cereus in vitro*. Effects on preformed biofilms were not investigated. This study represents a continuation of our research on the bioactivity of aronia [17,18].

## 2. Results and Discussion

**Figure 1:**
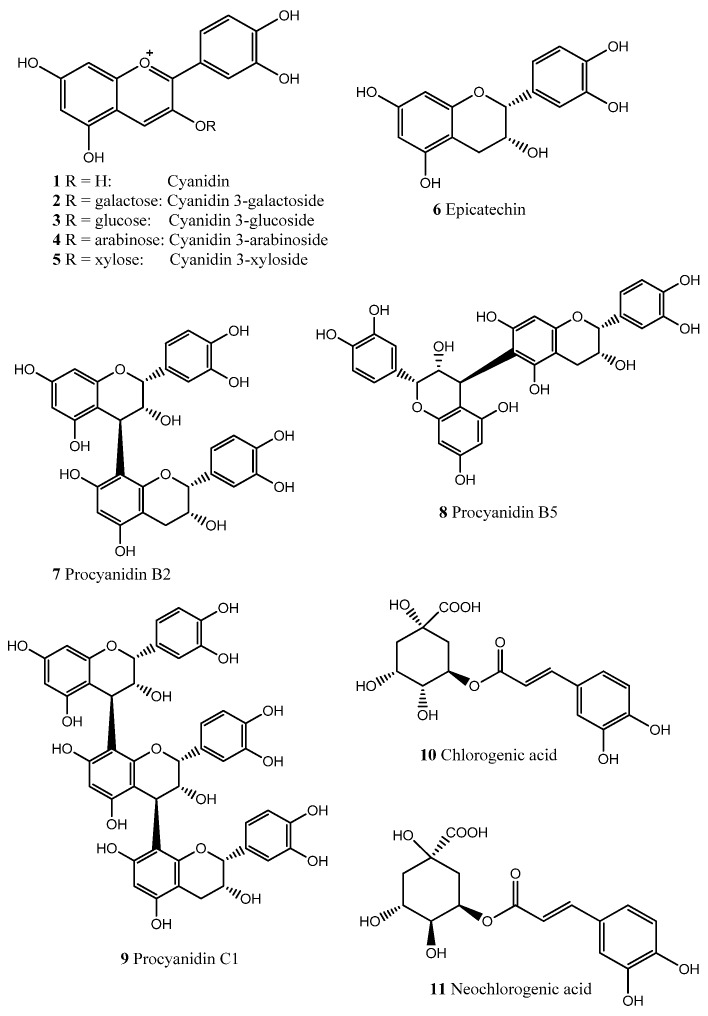
Chemical structures of compounds **1**–**11**.

Aronia berries were extracted with dichloromethane (DCM), 96% EtOH, 50% EtOH, and H_2_O as previously described [17]. ^1^H-NMR and ^13^C-NMR analyses revealed that proanthocyanidins were present in the 50% EtOH extract, which was further fractionated on a Sephadex LH-20 column to yield subfractions Seph a–g. Seph d and Seph g were chosen for further studies since their proanthocyanidin compositions had been well characterized. Seph d contained only trace amounts of proanthocyanidins with an average degree of polymerization (DP) of seven, whereas the majority of proanthocyanidins, with an average DP of 34, was found in Seph g. The proanthocyanidins in the subfractions were found to contain epicatechin as the monomeric unit. Also, compounds **1**–**11**, all well known constituents in aronia berries [14,15], were included in this study (Figure 1). Isolated compounds were identified on the basis of their chromatographic and spectroscopic data (TLC, HPLC, LC-MS) and optical rotations [17].

**Figure 2:**
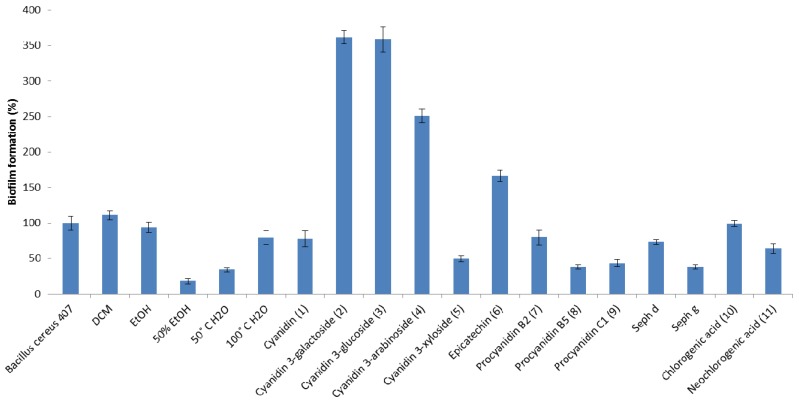
Biofilm formation of *B. cereus* strain 407 incubated with crude extracts, subfractions and compounds from aronia, compared to a non-treated control (*Bacillus cereus* 407, first bar from left) for which average biofilm formation was set to 100%.

**Figure 3:**
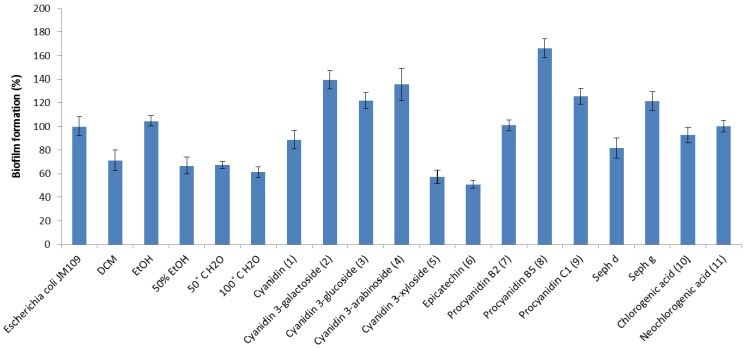
Biofilm formation of *E. coli* JM109 incubated with crude extracts, subfractions and compounds from aronia, compared to a non-treated control (*Escherichia coli* JM109, first bar from left) for which average biofilm formation was set to 100%.

Due to the growing interest in antimicrobial agents that can prevent or treat infections caused by biofilm-forming bacteria, aronia extracts, subfractions and compounds were tested for their ability to prevent biofilm production of two clinically relevant bacterial pathogens, *B. cereus* and *E. coli*, at sample concentrations of 1 mg/mL (125 µg per well) in a microtiter plate assay that constitutes a long-established method of measuring biofilm formation *in vitro* [19,20]. Maximum biofilm mass employing this assay was observed after 48 h of incubation for *B. cereus* [21] and after 24 h for *E. coli* (data not shown). These incubation times were therefore applied to investigate the abilities of aronia samples to inhibit biofilm formation. Polyvinyl chloride (PVC) microtitre plates were chosen in this assay since this material is often used in the manufacturing of medical devices, such as catheters [22]. The majority of aronia samples displayed biofilm inhibition against the Gram-positive *B. cereus* strain 407 (Figure 2), but exhibited less activity against Gram-negative *E. coli* JM109 (Figure 3). However, two samples (DCM extract and compound **6**) that showed no anti-biofilm activity against *B. cereus*, were effective against *E. coli*. In fact, compound **6** was the most active against biofilm-producing *E. coli*, whereas the 50% EtOH extract displayed the most potent inhibition of biofilm formation against *B. cereus*.

Among the anthocyanins, compounds **2**–**4** induced biofilm mass production of the bacteria, however, when *B. cereus* 407 and *E. coli* JM109 were grown in the presence of compound **5**, a 50% decrease in biofilm formation was observed. The cyanidin aglycone **1** possessed weak anti-biofilm activity against both bacteria. Thus, the ability to inhibit biofilm formation appears to be influenced by the sugar unit linked to the anthocyanidin. The largest variations in activity between the Gram-positive and Gram-negative bacterium were observed for the procyanidins. For *B. cereus*, monomeric epicatechin (**6**) increased biofilm mass, but dimeric (compounds **7** and **8**), trimeric (compound **9**) and polymeric (Seph d and Seph g) procyanidin fractions were effective in reducing biofilm production. Since procyanidin B5 (dimer), C1 (trimer) and fraction Seph g (polymer) appear about equally active, there is no clear correlation between degree of polymerization and inhibition of biofilm formation. On the contrary, monomeric epicatechin decreased biofilm mass of *E. coli*, whereas dimeric, trimeric and polymeric (except for Seph d) procyanidins increased its mass. Interestingly, we observed a considerable difference in anti-biofilm activity between procyanidin B2 (**7**) and the isomeric procyanidin B5 (**8**), indicating that minor structural differences might influence the ability to inhibit biofilm formation. Also, it was found that neochlorogenic acid (**11**) was more active as a biofilm inhibitor against *B. cereus* than the isomeric chlorogenic acid (**10**). However, their effects against *E. coli* were negligible.

The antibacterial activity of aronia substances against biofilm-forming *E. coli* JM109 (K12 strain) and *B. cereus* 407, and the uropathogenic *E. coli* strain CFT073 (which formed biofilm very poorly) was investigated by the disk diffusion method. Growth of the two *E. coli* strains was not affected by any aronia substance, and only 1.0 mg of the 50% EtOH extract showed some activity against *B. cereus* with a zone diameter of 10.2 ± 0.8 mm. However, it was much less efficient than the positive control gentamicin (10 µg) where a zone diameter of 20.5 ± 0.7 mm was observed. Hence, the inhibition of biofilm formation does not seem to be caused by toxicity towards the bacterial strains. Aronia samples might inhibit biofilm formation by interfering with quorum sensing, chemotaxis and/or motility genes that confer on cells a degree of motility in excess of that allowing adequate biofilm formation, as reported for *E. coli* K-12 exposed to ursolic acid by Ren *et al*. [1]. Investigation of the exact mechanism of this non-toxic inhibition, and also of potential effects of the compounds on already formed biofilms, would seem to be important subjects for further research. It has previously [2,23] been suggested that stress induced by the presence of the test substance might lead to increased production of EPS by the bacterial cell. This might be involved in the increase of biomass observed for some of the tested substances. At present, however, it is not clear why some of the test substances have this effect, while others do not. Conceivably, there may exist a balance between anti-biofilm activity (such as disturbance of quorum sensing) and biofilm stimulating activity, and as shown in our study, this balance would seem to be dependent on minor differences in chemical structure. In our experiments, blanks without bacteria added to the growth medium served as negative controls for biofilm formation. We also included controls without aronia sample added, as negative controls for biofilm inhibition. This study was designed to investigate inhibition of biofilm formation, starting out from planktonic cultures. Thus, no antibiotics were added to the assay system, as the sensitivity of the planktonic bacteria to the antibiotics would not allow growth and formation of a biofilm structure. Future experiments may include testing the chemotolerance of cells within a biofilm, by adding antibiotics to a pre-formed biofilm.

The prevalence of bacterial pathogens living in biofilms, where they are much more resistant to antibiotics and clearance by the immune system compared to planktonic cells, has promoted the search for new strategies to control biofilm infections [9]. Previous studies have included the influence of cranberry extracts on the formation of oral biofilms [24,25], on biofilm produced by *Staphylococcus epidermidis* on contact lenses [26], and by cranberry juice on biofilm produced by uropathogenic *E. coli* strains [27,28]. These studies confirm that cranberry juice can lead to a decrease in the ability of pathogenic bacteria to develop biofilm on inert surfaces such as urinary catheters and contact lenses. Cranberry contains A-type proanthocyanidins, which have been implicated as active constituents responsible for its bacterial anti-adhesive properties that prevent the attachment of *E. coli* onto uroepithelial cells, thereby preventing urinary tract infections [29]. However, it has previously been reported that cranberry juice is more effective than isolated cranberry proanthocyanidins alone in preventing biofilm formation [22,29]. In our study, the 50% EtOH extract was the most potent inhibitor of *B. cereus* biofilm formation. This might be due to the presence of unknown active compounds in this extract, or to synergistic effects. Against *E. coli* biofilm, epicatechin was the most effective substance tested, while effects of oligomeric and polymeric proanthocyanidins were negligible. Interestingly, cyanidin 3-xyloside showed activity against Gram-negative *E. coli* and Gram-positive *B. cereus*, whereas the other anthocyanins were inactive. Recently, a large number of flavonoids have been investigated for anti-biofilm activity towards *Staphylococcus aureus* [30]. In that study, flavones, chalcones, flavonols, flavans, flavanones, isoflavonoids, neoflavonoids and dihydroflavonols were studied. Our work on catechins, anthocyanins and procyanidins would therefore seem to extend the range of active flavonoids. The results presented underscore the need for more research on the anti-biofilm effect of berries and their constituents. This work was intended to investigate whether aronia extracts and constituents can inhibit formation of biofilms. Other aspects such as study of other endpoints than biomass, dose-response relationships, and bacterial viability would be highly relevant subjects for future research.

## 3. Experimental

### 3.1. General

Two Gram-negative *Escherichia coli* strains (*E. coli* K12 JM109, and uropathogenic *E. coli* CFT073 (ATCC 700928)) and one Gram-positive *Bacillus cereus* strain (*B. cereus* 407 wild-type [31]) were used in the present study. For each organism, frozen glycerol (20%) stocks in lysogeny broth (LB) medium were prepared and maintained at −80 °C. A HTS 7000 Plus Bio Assay Reader (Perkin Elmer, Waltham, MA, USA) was used for UV-Vis measurements during the biofilm screening procedure. Gentamicin antimicrobial susceptibility test disks were obtained from Oxoid Ltd., (Basingstoke, UK). Epicatechin (**6**), chlorogenic acid (**10**) and neochlorogenic acid (**11**) were purchased from Sigma-Aldrich (St. Louis, MO, USA).

### 3.2. Plant Material

Aronia berries (*Aronia melanocarpa* (Michx.) Elliott var. Moscow (Rosaceae)) were harvested at Bioforsk Vest Særheim, Klepp, Norway (58°47'N, 5°41'E) in August 2010 and identified by one of the authors (R. Slimestad). The berries were kept at −20 °C until extraction. A voucher specimen (MB201201) is deposited in the Pharmacognosy section, School of Pharmacy, University of Oslo, Norway. Bark, as source of procyanidins, was sampled from the same plants. Branches with a diameter of 1–2 cm were chosen, and the bark was carefully removed. The plant material was cut in pieces and kept at −20 °C until extraction.

### 3.3. Extraction, Fractionation and Isolation

Aronia berries were extracted with DCM and 96% EtOH, 50% EtOH, and H_2_O followed by chromatography of the 50% EtOH extract to give subfractions Seph a–g, as previously described [17]. In parallel to this, cyanidin aglycone (**1**), four anthocyanins: cyanidin 3-galactoside (**2**), cyanidin 3-glucoside (**3**), cyanidin 3-arabinoside (**4**) and cyanidin 3-xyloside (**5**) and three procyanidins: procyanidin B2 (**7**), procyanidin B5 (**8**) and procyanidin C1 (**9**) were isolated from aronia berries (compounds **1**–**5**) and bark (compounds **7**–**9**). The detailed protocol for compound purification has been described previously [18]. Cyanidin was prepared by acidic hydrolysis of cyanidin 3-galactoside. Cyanidin 3-galactoside (about 100 mg) was dissolved in MeOH (0.5% HCl, 5 mL) and mixed with 2 M HCl (5 mL). Hydrolysis occurred in a capped glass tube at 100 °C for 30 min. Cyanidin was isolated from the mixture by elution with 50% MeOH (0.1% HCl) over a bed of Sephadex LH-20 (3 × 40 cm Pyrex column). Purity was determined to be above 97% by HPLC [18].

### 3.4. Microtiter Plate Biofilm Formation Assay

Biofilm screening was performed essentially as in Auger *et al*. [4], with some modifications. Microorganisms were streaked from frozen glycerol stocks (−80 °C) onto LB agar plates and incubated overnight (30 °C for *B. cereus*, 37 °C for *E. coli*). After growth on solid medium, an isolated colony was picked and inoculated in 5 mL LB medium for 18 h, at 30 °C for *B. cereus* and 37 °C for *E. coli*, under constant agitation at 225 rpm. Precultures were prepared by inoculating 50 µL of the 18 h culture in 5 mL LB medium (1:100), and incubated as before for 3 h. Further, 6 µL of the preculture was diluted in 1 mL fresh bactopeptone medium and 200 µL of test substance (dissolved in H_2_O or 10% MeOH in H_2_O) was added. 125 µL of each diluted culture was transferred to eight wells of a 96-well PVC microtiter plate (Falcon 353911). In each plate, eight wells were used as blanks, containing bactopeptone medium only. For coloured samples, blanks contained bactopeptone medium and the respective substances. After incubation for 48 h at 30 °C for *B. cereus* and 24 h at 37 °C for *E. coli*, which constituted the incubation times for which biofilm formation had been determined to be at its maximum for the given experimental set up, the biofilm density was measured as follows: the microtiter plate wells were washed once with phosphate-buffered saline (PBS) in order to remove non-adherent bacteria. Bacterial cells bound to the walls of the wells were stained with a 1% (w/v) crystal violet solution at room temperature for 20 min. The wells were then washed three times with PBS, followed by solubilization of the dye in an acetone/ethanol (1:4) mixture by slowly and continuously pipetting up and down three times to ensure extraction of the dye from cells. The mixture was immediately transferred to a transparent flat-bottomed microtiter plate and the absorbance at 492 nm of the solubilized dye was subsequently determined. *E. coli* K12 JM109 and *B. cereus* 407 were good biofilm-forming strains. Uropathogenic *E. coli* CFT073 was tested, but made a very poor biofilm under the same conditions. Screening studies of the various test samples on biofilm inhibition were carried out as three individual experiments, with eight technical replicates for each experiment, and the results are presented as averages ± SEM. Test sample concentrations of 1 mg/mL (125 µg per well) were applied. Samples dissolved in 10% MeOH in H_2_O had a total MeOH concentration of 1.7% during incubation.

### 3.5. Antimicrobial Disk Susceptibility Test

Antimicrobial activity was determined according to the guideline *Performance Standards for Antimicrobial Disk Susceptibility Tests; Approved Standard—Ninth Edition* (CLSI document M2-A9) [32]. A small amount of isolated colonies from 18 to 24 h old pure culture was suspended in sterile saline and the turbidity of this suspension was adjusted to a 0.5 McFarland standard. A dipped swab was then used to inoculate a Mueller-Hinton agar plate. Antimicrobial susceptibility blank disks (BBL, 6 mm in diameter), containing either 1.0 mg (50 µL, 20 mg/mL) and 125 µg (50 µL, 2.5 mg/mL) crude extract or 125 µg of compounds **1**–**11** and fractions Seph d and Seph g, were placed on the agar surface together with a positive control disk (gentamicin 10 µg) and a negative control disk containing 50 µL solvent (H_2_O or MeOH). Culture plates were incubated for 18 h at 37 °C, and zone diameters were recorded. All tests were performed in triplicate.

## 4. Conclusions

The presence of biofilms on medical devices and their role in infections is well known. The tolerance of catheter-associated biofilm-forming organisms toward antimicrobial treatments in the health care environment underscores the importance of alternative treatment strategies. In the present study, we have shown that exposure of biofilm-forming *E. coli* and *B. cereus* strains to several aronia constituents reduced biofilm production. Whether our results can be used in future development of biofilm inhibitors requires further investigation.
